# Different actions of endothelin-1 on chemokine production in rat cultured astrocytes: reduction of CX3CL1/fractalkine and an increase in CCL2/MCP-1 and CXCL1/CINC-1

**DOI:** 10.1186/1742-2094-10-51

**Published:** 2013-04-30

**Authors:** Yutaka Koyama, Mao Kotani, Tadateru Sawamura, Miho Kuribayashi, Rika Konishi, Shotaro Michinaga

**Affiliations:** 1 Laboratory of Pharmacology, Faculty of Pharmacy, Osaka Ohtani University, 3-11-1 Nishikiori-Kita, Tonda-bayashi, Osaka, 584-8540, Japan

**Keywords:** Endothelin-1, Brain injury, Chemokines, Gene expression

## Abstract

**Background:**

Chemokines are involved in many pathological responses of the brain. Astrocytes produce various chemokines in brain disorders, but little is known about the factors that regulate astrocytic chemokine production. Endothelins (ETs) have been shown to regulate astrocytic functions through ET_B_ receptors. In this study, the effects of ETs on chemokine production were examined in rat cerebral cultured astrocytes.

**Methods:**

Astrocytes were prepared from the cerebra of one- to two-day-old Wistar rats and cultured in serum-containing medium. After serum-starvation for 48 hours, astrocytes were treated with ETs. Total RNA was extracted using an acid-phenol method and expression of chemokine mRNAs was determined by quantitative RT-PCR. The release of chemokines was measured by ELISA.

**Results:**

Treatment of cultured astrocytes with ET-1 and Ala^1,3,11,15^-ET-1, an ET_B_ agonist, increased mRNA levels of CCL2/MCP1 and CXCL1/CINC-1. In contrast, CX3CL1/fractalkine mRNA expression decreased in the presence of ET-1 and Ala^1,3,11,15^-ET-1. The effect of ET-1 on chemokine mRNA expression was inhibited by BQ788, an ET_B_ antagonist. ET-1 increased CCL2 and CXCL1 release from cultured astrocytes, but decreased that of CX3CL1. The increase in CCL2 and CXCL1 expression by ET-1 was inhibited by actinomycin D, pyrrolidine dithiocarbamate, SN50, mithramycin, SB203580 and SP600125. The decrease in CX3CL1 expression by ET-1 was inhibited by cycloheximide, Ca^2+^ chelation and staurosporine.

**Conclusion:**

These findings suggest that ETs are one of the factors regulating astrocytic chemokine production. Astrocyte-derived chemokines are involved in pathophysiological responses of neurons and microglia. Therefore, the ET-induced alterations of astrocytic chemokine production are of pathophysiological significance in damaged brains.

## Background

Chemokines were originally identified as a family of small proteins having chemoattractive activities on inflammatory cells. Various chemokines are constitutively or inducibly expressed in the brain and are involved in physiological or pathological nerve functions [1,2]. In brain ischemia, head trauma and neurodegenerative diseases, the expression of brain chemokines is altered, which modulates neuroinflammation and the repair process of damaged nerve tissues [3]. Astrocytes are one of the chemokine-producing cells in the brain. Immunohistochemical observations on damaged nerve tissues showed that production of brain chemokines, including CCL2/monocyte chemoattractant protein-1 (MCP-1) and CXCL1/cytokine-induced neutrophil chemoattractant-1 (CINC-1), increased in astrocytes [4-6]. Astrocyte-derived chemokines act on brain microvascular endothelial cells. The chemokine-induced functional changes of vascular endothelial cells promote infiltration of inflammatory cells and neovascularization at the damaged areas. In addition to vascular endothelial cells, expression of chemokine receptors in normal and pathological brains were shown in neurons, astrocytes and microglia [1], suggesting that the function of these brain cells is also regulated by chemokines. During brain injury, the production of astrocyte-derived extracellular signal molecules affects the viability of damaged neurons and the repair of nerve tissues [7]. Studies in cultured neurons showed that some types of chemokines had a protective effect against neuronal damage, while other types were detrimental [8-12]. In brain disorders, microglia become activated. Astrocytes are also involved in the regulation of microglial activation by releasing signal molecules [7]. Microglial activation is accompanied by the enhancement of microglial function, including phagocytosis, migration and pro-inflammatory cytokine production. *In vitro* and *in vivo* studies showed that these microglial functions are modulated by certain chemokines [13-19]. From the various actions of brain chemokines, important roles of astrocytic chemokine production in neuroinflammation and the tissue repair process after brain injury are proposed. However, the regulatory mechanisms of chemokine production in astrocytes are not fully understood.

Endothelins (ETs), a vasoconstrictor peptide family, are present in the brain. The production of brain ETs is increased in various brain disorders. Increases in brain ETs are involved in the pathophysiological responses of nerve tissues [20-22]. Receptors for ETs are classified as ET_A_ or ET_B_ types. In the brain, high expression of ET_B_ receptors was observed in astrocytes [23,24]. ETs have been shown to regulate the function of astrocytes through ET_B_ receptors. In animal brain injury models, ET_B_ antagonists reduced astrocytic proliferation [25,26], indicating that ET_B_ receptors are involved in the induction of astrogliosis. Activation of ET_B_ receptors was shown to induce the production of several signaling molecules, such as neurotrophic factors and cytokines, in cultured astrocytes and in the rat brain [27]. These findings suggest that ETs regulate the pathophysiological response of the damaged brain by modulating the production of astrocytic signaling molecules. As for the production of chemokines in the brain, we previously showed that administration of an ET_B_ agonist increased CCL2 and CXCL1 production in the adult rat brain [28]. In this study, to clarify the role of ET_B_ receptors in astrocytic chemokine production, the effect of ETs on chemokine expression in rat cultured astrocytes was examined.

## Methods

### Preparation of rat primary cultured astrocytes

All experimental protocols conformed to the Guiding Principles for the Care and Use of Animals of the Japanese Pharmacological Society and were approved by the Animal Experiment Committee of Osaka Ohtani University. Astrocytes were prepared from the cerebra of one- to two-day-old Wistar rats as described previously [29]. The isolated cells were seeded at 1 × 10^4^ cells/cm^2^ in 75-cm^2^ culture flasks and grown in minimal essential medium (MEM) supplemented with 10% fetal calf serum. To remove small process-bearing cells (mainly oligodendrocyte progenitors and microglia from the protoplasmic cell layer), the culture flasks were shaken at 250 rpm overnight, 10 to 14 days after seeding. The monolayer cells were trypsinized and seeded on six-well culture plates. Astrocytes were identified by immunocytochemical observations of glial fibrillary acidic protein (GFAP), an astrocytic marker protein. At this stage, approximately 95% of cells showed immunoreactivity for GFAP. Cultured neurons and microglia were prepared from the rat cerebrum according to previously described methods [29].

### Treatment with ETs and the other drugs

Before treatment with ETs and other drugs, astrocytes in six-well culture plates were cultured in serum-free MEM for 48 hours. ET-1 and Ala^1,3,11,15^-ET-1 were dissolved in distilled H_2_O to make stock solutions. ET antagonists and signal transduction inhibitors were dissolved in dimethyl sulfoxide (DMSO). Treatments of cultured astrocytes with ETs and other drugs were started by addition of the stock solutions to serum-free MEM. As a control for treatments with ET antagonists and signal transduction inhibitors, equal volumes of DMSO were included in the medium.

### Measurement of chemokine mRNA levels by quantitative RT-PCR

Total RNA was extracted using an acid-phenol method as described previously [29]. First-strand cDNA was synthesized from total RNA (1 μg) using MMLV reverse transcriptase (200 U; Invitrogen, Carlsbad, CA, USA), random hexanucleotides (0.2 μg; Invitrogen) and an RNase inhibitor (20 U; Takara, Tokyo, Japan) in 10 μL of buffer supplied by the enzyme manufacturer. The mRNA levels of chemokines in each sample were determined by quantitative PCR using SYBR Green fluorescent probes. Each reverse transcription product was added to the SYBR Green Master Mix (Toyobo, Tokyo, Japan) along with the primer pairs, and the mixture was placed in a thermal cycler (Opticom 2; MJ Research, Waltham, MA, USA). The following primer pairs were used:

CCL2,

5′-TTCACTGGCAAGATGATCCC-3′ and 5′-TGCTTGAGGTGGTTGTGGAA-3′;

CXCL1,

5′- GAAGATAGATTGCACCGATG -3′ and 5′- CATAGCCTCTCACACATTTC-3′;

CCL5/RANTES,

5′-CACCGTCATCCTCGTTGC-3′ and 5′-CACTTGGCGGTTCCTTCG-3′;

CX3CL1/fractalkine,

5′- GAATTCCTGGCGGGTCAGCACCTCGGCATA-3′ and 5′-AAGCTTTTACAGGGCAGCCGTCTGGTGGT-3′

CXCL12/ stromal cell-derived factor-1 (SDF-1),

5′-TTGCCAGCACAAAGACACTCC-3′ and 5′-CTCCAAAGCAAACCGAATACAG-3′;

glyceraldehydes-3-phosphate dehydrogenase (G3PDH),

5′-CTCATGACCACAGTCCATGC-3′ and 5′-TACATTGGGGGTAGGAACAC-3′.

As a standard for the copy number of PCR products, serial dilutions of each amplicon were amplified in the same manner. The amount of cDNA was calculated as the copy number of each reverse-transcription product equivalent to 1 μg of total RNA and normalized to the value for G3PDH.

### Determination of chemokine proteins

Serum-starved astrocytes in six-well plates were treated with ET-1 and the culture medium collected. The level of immunoreactive chemokines in the culture media were determined using an ELISA kit for rat CCL2 (Biosource, Camarillo, CA, USA), CXCL1 (Immuno-Biological Laboratories, Gunma, Japan,) and CX3CL1 (RayBiotech, Norcross, GA, USA) according to the manufacturers’ protocols. The protein content in each well was determined with a BCA protein assay kit (Pierce, Rockford, IL, USA).

## Results

### Effect of ETs on chemokine production in cultured astrocytes

In the adult rat brain, the mRNA of CCL2, CXCL1, CCL5/RANTES, CX3CL1/fractalkine and CXCL12/SDF-1 has been previously detected [4,6,30-32]. These chemokines are produced by different brain cells, including neurons, microglia and astrocytes [3]. Thus, at first, copy numbers of these chemokine mRNAs in cultured neurons, microglia and astrocytes were determined. Copy numbers of CCL2 and CXCL1 in non-stimulated cultured astrocytes were 10 to 50 times higher than those in neurons and microglia (Table 1). Expression of CX3CL1 was high in neurons and astrocytes. Copy numbers of CXCL12 and RANTES were of similar level among these cells.

**Table 1:** Comparison of chemokine mRNA copy numbers in neurons, astrocytes and microglia derived from the rat cerebrum

**mRNA copy number**			
**(× 10**^**3**^**/μg total RNA)**			
	**Neuron**	**Astrocyte**	**Microglia**
CCL2/MCP-1	189.9 ± 9.8	3050.8 ± 521.5	148.9 ± 31.6
CCL5/RANTES	10.3 ± 0.7	52.5 ± 18.7	4.3 ± 2.2
CXCL1/CINC-1	22.0 ± 2.2	1410.0 ± 266.9	34.4 ± 18.1
CXCL12/SDF-1α	92.1 ± 4.0	255.4 ± 45.7	29.1 ± 13.6
CX3CL1/fractalkine	1,118.1 ± 108.4	1,721.0 ± 303.5	23.4 ± 6.6
G3PDH	40,081.9 ± 7,335.2	46,575.5 ± 6,274.4	48,586.5 ± 5,733.6

Cultured neurons, astrocytes and microglia were prepared from the cerebra of Wistar rats and total RNA was extracted. The copy numbers of chemokine mRNA were determined by quantitative RT-PCR. The copy numbers of G3PDH mRNA in the same samples were also determined as an internal standard. Data are the mean ± SEM of 6 to 10 different preparations presented as ×10^3^ copy numbers/μg total RNA. G3PDH, glyceraldehyde-3-phosphate dehydrogenase; SEM, standard error of the mean.

**Figure 1:**
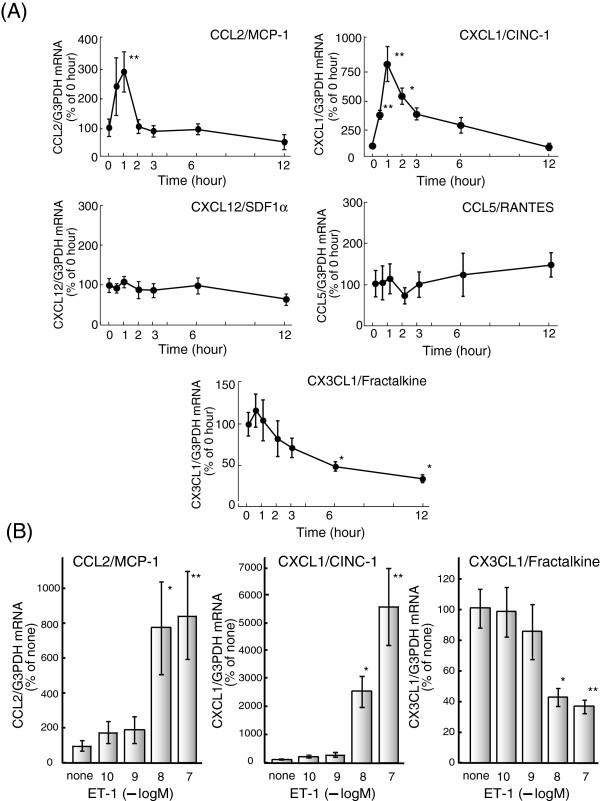
**Effect of ET-1 on chemokine mRNA expression in cultured rat astrocytes.** (**A**) Serum-starved astrocytes were treated with 100 nM ET-1 for the times indicated. The expression of CCL2, CXCL1, CCL5, CXCL12 and CX3CL1 mRNA was normalized to G3PDH and expressed as the % of 0 hour. Data are the mean ± SEM of 6 to 16 experiments. *P <0.05 and **P <0.01 *versus* 0 hour by one-way ANOVA followed by Dunnett’s test. (**B**) Astrocytes were treated with the indicated concentrations of ET-1 for one (CCL2 and CXCL1) or six (CX3CL1) hours. Data are the mean ± SEM of five to eight experiments. *P <0.05 and **P <0.01 *versus* none by one-way ANOVA followed by Dunnett’s test. ANOVA, analysis of variance; ET-1, endothelin-1; G3PDH, glyceraldehyde-3-phosphate dehydrogenase; SEM, standard error of the mean.

**Figure 2:**
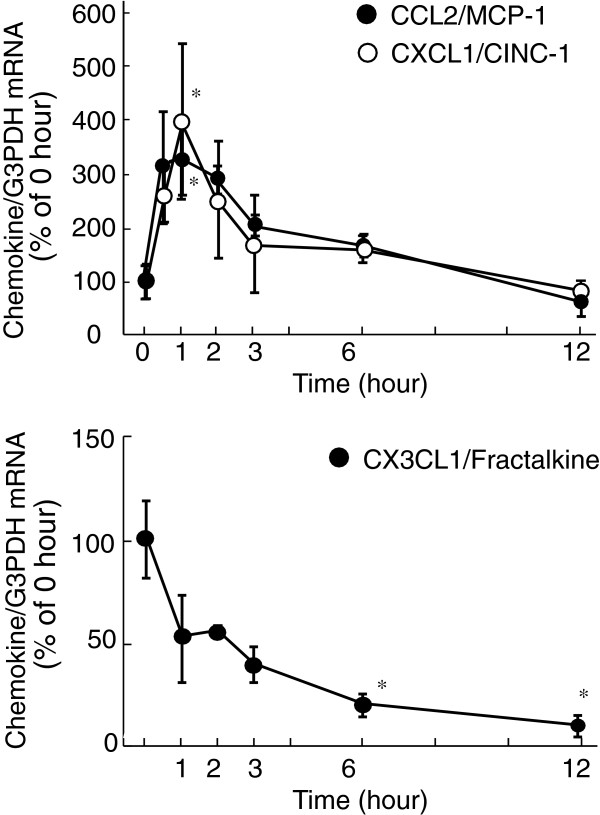
**Effects of Ala**^**1,3,11,15**^**-ET-1 on CCL2, CXCL1 and CX3CL1 mRNA expression in cultured rat astrocytes.** Serum-starved astrocytes were treated with 100 nM Ala^1,3,11,15^-ET-1 for the times indicated. The expression of CCL2, CXCL1 and CX3CL1 mRNA was normalized to G3PDH and expressed as the % of 0 hour. Data are expressed as the mean ± SEM of 4 to 14 experiments. *P <0.05 *versus* 0 hour by one-way ANOVA followed by Dunnett’s test. ANOVA, analysis of variance; ET-1, endothelin-1; G3PDH, glyceraldehyde-3-phosphate dehydrogenase; SEM, standard error of the mean.

**Figure 3:**
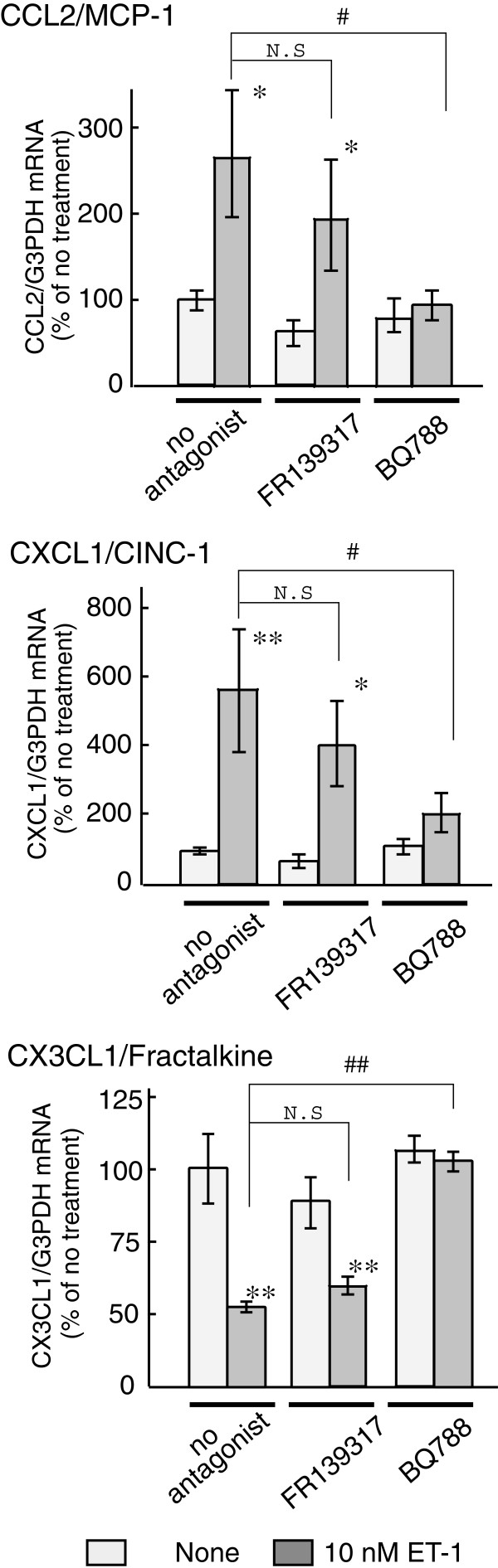
**Effects of ET receptor antagonists on ET-induced changes in CCL2, CXCL1 and CX3CL1 mRNA levels.** Serum-starved astrocytes were treated with 10 nM ET-1 for one (CCL2 and CXCL1) or six (CX3CL1) hours**.** BQ788 (1 μM) or FR139317 (1 μM) was added to the medium 30 minutes before treatment with ET-1. The expression of CCL2, CXCL1 and CX3CL1 mRNA was normalized to G3PDH and expressed as the % of no treatment cultures. Data are the mean ± SEM of five to nine experiments. *P <0.05, **P <0.01 *versus* no ET-1, ^#^*P* <0.05, ^##^*P* <0.01 *versus* ET-1 with no antagonist by one-way ANOVA followed by Fisher’s PLSD test. ANOVA, analysis of variance; ET, endothelin; G3PDH, glyceraldehyde-3-phosphate dehydrogenase; NS, not significant; PLSD, protected least significant difference; SEM, standard error of the mean.

**Figure 4:**
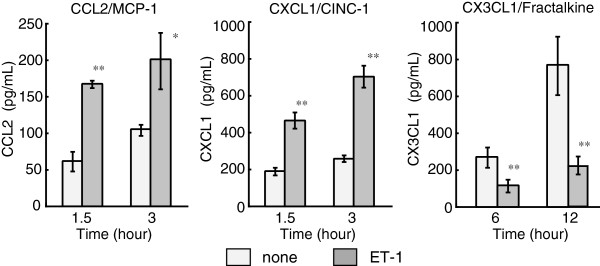
**Effect of ET-1 on immunoreactive CCL2, CXCL1 and CX3CL1 released from cultured astrocytes.** Astrocytes were cultured in serum-free MEM in the absence or presence of 100 nM ET-1 for the times indicated. Concentrations of immunoreactive CCL2, CXCL1 and CX3CL1 in the culture medium were measured by ELISA. Results are the mean ± SEM of six to eight experiments. *P <0.05, **P <0.01 *versus* no ET-1 by one-way ANOVA followed by Fisher’s PLSD test. ANOVA, analysis of variance; ET, endothelin; MEM, minimal essential medium; PLSD, protected least significant difference; SEM, standard error of the mean.

Treatment of cultured astrocytes with 100 nM ET-1 increased mRNA levels of CCL2 and CXCL1, where the maximum increase was observed in one hour (Figure 1A). In contrast, CX3CL1 mRNA decreased following ET-1 exposure to approximately 40% of levels observed in non-treated cells in six hours. ET-1 did not affect mRNA levels of CCL5 and CXCL12. The effect of ET-1 on CCL2 and CXCL1 mRNA levels was dose-dependent and a significant increase was observed at 10 nM (Figure 1B). The ET-induced decrease in astrocytic CX3CL1 mRNA was significant at 10 nM. Treatment with 100 nM Ala^1,3,11,15^-ET-1, a selective ET_B_ agonist, also increased CCL2 and CXCL1 mRNA levels in cultured astrocytes, while it decreased CX3CL1 mRNA (Figure 2). Increases in CCL2 and CXCL1 mRNA by ET-1 were inhibited by 1 μM BQ788, an ET_B_ antagonist (Figure 3). BQ788 also inhibited the ET-induced decrease in CX3CL1 mRNA. FR139317 (1 μM), an ET_A_ antagonist, did not inhibit the effects of ET-1 on astrocytic CCL2, CXCL1 and CX3CL1 mRNA levels. The effects of ET-1 on chemokine release from cultured astrocytes were examined. Treatment with 100 nM ET-1 for 1.5 to 3 hours increased the release of CCL2 and CXCL1 protein in the culture medium, while release of CX3CL1 protein into the culture medium decreased in the presence of ET-1 (Figure 4).

### Effects of signal transduction inhibitors on the ET-induced alterations of chemokine production

**Figure 5:**
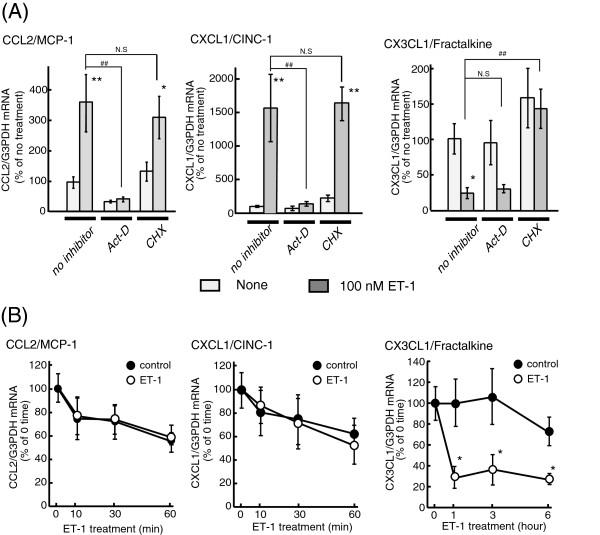
**Effects of actinomycin D and cycloheximide on ET-induced changes in CCL2, CXCL1 and CX3CL1 mRNA levels.** (**A**) Serum-starved astrocytes were treated with 100 nM ET-1 for one (CCL2 and CXCL1) or six (CX3CL1) h**ours.** Actinomycin D (Act-D, 1 μg/mL) or cycloheximide (CHX, 10 μg/mL) was added to the medium 30 minutes before treatment with ET-1. The expression of CCL2, CXCL1 and CX3CL1 mRNA was normalized to G3PDH and expressed as the % of no treatment cultures. Data are the mean ± SEM of 8 to 15 experiments. *P <0.05, **P <0.01 *versus* no ET-1, ^##^*P* <0.01 *versus* no inhibitor by one-way ANOVA followed by Fisher’s PLSD test. NS, not significant. (**B**) Effects of ET-1 on CCL2, CXCL1 and CX3CL1 mRNA expressions in the presence of actinomycin D. Cultured astrocytes were treated with ( open circle) or without (closed circle) 100 nM ET-1 for the time indicated in the presence of 1 μg/mL actinomycin D. Actinomycin D was included in the medium 30 minutes before treatment with ET-1. Data are the mean ± SEM of six experiments. *P <0.05 *versus* control by one-way ANOVA followed by Fisher’s PLSD test. ANOVA, analysis of variance; ET-1, endothelin-1; G3PDH, glyceraldehyde-3-phosphate dehydrogenase; PLSD, protected least significant difference; SEM, standard error of the mean.

The expression of the mRNAs of several chemokines is regulated by transcriptional mechanisms and alterations of mRNA stability. Involvement of transcriptional mechanisms in the ET-induced alterations of astrocytic chemokine production were examined by using actinomycin D, a transcription inhibitor. Actinomycin D (1 μg/mL) gradually decreased basal expressions of CCL2 and CXCL1 mRNAs in the treatments up to 60 minutes, although the effects were not statistically significant (Figure 5A and B). In the presence of actinomycin D, ET-1 did not increase astrocytic CCL2 and CXCL1 mRNAs (Figure 5A and B). On the other hand, the ET-induced decrease in CX3CL1 expression was not affected by actinomycin D (Figure 5A and B). Cycloheximide (10 μg/mL), a protein synthesis inhibitor, had no effect on ET-induced CCL2 and CXCL1 expression, but prevented the decrease in CX3CL1 expression (Figure 5A).

**Figure 6:**
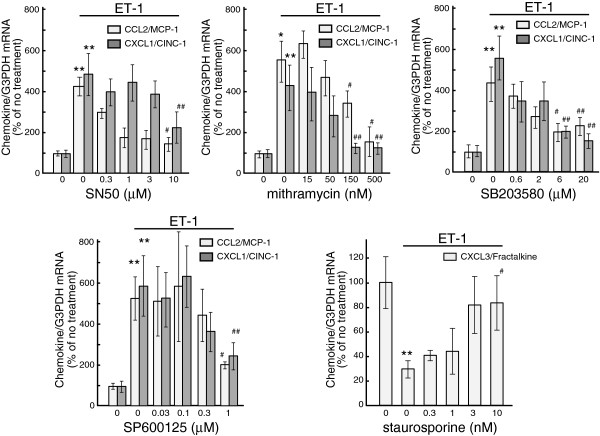
**Dose-dependent inhibition of ET-induced changes in CCL2, CXCL1 and CX3CL1 mRNA levels by signal transduction inhibitors.** Serum-starved astrocytes were treated with 100 nM ET-1 for one (CCL2 and CXCL1) or six (CX3CL1) h**ours.** Different concentrations of signal transduction inhibitors (SN50, mithramycin, SB203580, SP600125 and staurosporine) were included in the medium 30 minutes before treatment with ET-1. The expression of CCL2, CXCL1 and CX3CL1 mRNA was normalized to G3PDH and expressed as the % of no treatment cultures. Data are the mean ± SEM of 6 to 12 experiments. *P <0.05, **P <0.01 *versus* no treatment, ^#^*P* <0.05, ^##^*P* <0.01 *versus* ET-1 with no inhibitor by one-way ANOVA followed by Fisher’s PLSD test. ANOVA, analysis of variance; ET, endothelin; G3PDH, glyceraldehyde-3-phosphate dehydrogenase; PLSD, protected least significant difference; SEM, standard error of the mean.

ET_B_ receptors belong to Gq-protein coupled receptors. Activation of astrocytic ET_B_ receptors induces an increase in cytosolic Ca^2+^ and activation of protein kinase C (PKC) and mitogen-activated protein (MAP) kinases [25,33-35]. Ca^2+^ chelation (a combination of 0.5 mM ethylene glycol tetraacetic acid (EGTA) and 30 μM 1,2-bis(2-aminophenoxy)ethane N,N,N',N'-tetraacetic acid acetoxymethyl ester. (BAPTA/AM)) and PKC inhibition (staurosporine, 10 nM) did not affect ET-induced CCL2 and CXCL1 mRNA expression (Table 2). On the other hand, the decrease in CX3CL1 expression was inhibited by Ca^2+^ chelation and staurosporine. The inhibition by staurosporine was BAPTA/AM dose-dependent, where a significant effect was obtained at 10 nM (Figure 6). SB203580 (a p38 inhibitor) and SP600125 (a JNK inhibitor) inhibited the effect of ET-1 on CCL2 and CXCL1 expression in a dose-dependent manner, but PD98059 (an ERK inhibitor, 50 μM) had no effect (Table 2 and Figure 6). The ET-induced decrease in CX3CL1 expression was not affected by these MAP kinase inhibitors (Table 2). Pyrrolidine dithiocarbamate (PDTC, 100 μM) and SN50 (10 μM), which inhibits the transcriptional activities of nuclear factor-kappaB (NFκB), reduced ET-induced CCL2 and CXCL1 expressions, while these inhibitors did not alter the effects of ET-1 on CX3CL1 expression (Table 2 and Figure 6). Mithramycin (500 nM), an inhibitor of transcription factor SP1, diminished ET-induced CCL2 and CXCL1 expression, but had no effect on the decrease of CX3CL1 expression (Table 2 and Figure 6). At the highest concentrations used, these signal transduction inhibitors did not largely affect basal expressions of astrocytic CCL2, CXCL1 and CX3CL1 mRNAs [see Additional file 1].

**Table 2:** Effect of signal transduction inhibitors on ET-induced expression of CCL2, CXCL1 and CX3CL1 mRNA

	**Ratio of chemokine to G3PDH mRNA copy number**		
	**(% of no treatment)**		
	**CCL2/MCP-1**	**CXCL1/CINC-1**	**CX3CL1/fractalkine**
no treatment	100.0 ± 31.4 (20)	100.0 ± 22.0 (20)	100.0 ± 17.5 (24)
100 nM ET-1	313.8 ± 65.1 (20)^a^	404.7 ± 63.9 (20)^a^	37.5 ± 8.5 (23)^a^
+ 30 μM BAPTA/0.5 mM EGTA	307.4 ± 89.0 (7)	342.7 ± 65.0 (7)	122.8 ± 25.2 (9)^b^
+ 10 nM staurosporine	444.8 ± 122.3 (6)	385.4 ± 79.0 (6)	108.6 ± 25.6 (4)^b^
+ 100 μM PDTC	129.2 ± 16.5 (12)^b^	140.2 ± 20.7 (12)^b^	42.5 ± 8.8 (18)
+ 10 μM SN50	188.7 ± 64.3(8)^b^	136.1 ± 22.6 (8)^b^	54.6 ± 23.9 (11)
+ 500 nM mithramycin	37.8 ± 9.2 (8)^c^	49.8 ± 24.1 (8)^c^	36.3 ± 11.7 (10)
+ 50 μM PD98059	277.4 ± 90.7 (12)	360.2 ± 103.2 (12)	49.6 ± 7.2 (10)
+ 20 μM SB203580	118.3 ± 36.9 (13)^c^	229.1 ± 73.6 (13)^b^	37.1 ± 6.3 (20)
+ 1 μM SP600125	110.2 ± 23.6 (8)^b^	188.5 ± 22.5 (8)^b^	47.1 ± 22.0 (10)

^a^*P* <0.01 *versus* no treatment, ^b^*P* <0.05, ^c^*P* <0.01, *versus* 100 nM ET-1 by one-way ANOVA followed by Fisher’s PLSD test. Astrocytes were treated with 100 nM ET-1 in the presence of the signal transduction inhibitors indicated. Total RNA was extracted at one hour (for CCL2 and CXCL1) or six hours (for CX3CL1) after the addition of ET-1. The inhibitors were included in the serum-free medium for 30 minutes before the addition of ET-1. The copy numbers of CCL2, CXCL1 and CX3CL1 mRNA was normalized to G3PDH. Results are the mean ± SEM and the numbers of experiments are indicated in parentheses. ANOVA, analysis of variance; BAPTA/AM, 1,2-bis(2-aminophenoxy)ethane N,N,N',N'-tetraacetic acid acetoxymethyl ester; ET, endothelin; G3PDH, glyceraldehyde-3-phosphate dehydrogenase; PDCT, pyrrolidine dithiocarbamate; PLSD, protected least significant difference; SEM, standard error of the mean.

## Discussion

### ETs increase astrocytic CCL2 and CXCL1 production

Various chemokines, including CCL2, CXCL1, CCL5, CXCL12 and CX3CL1, are constitutively or inducibly expressed in the adult brain. A comparison of these chemokine mRNA levels in cultured neurons, microglia and astrocytes (Table 1) revealed higher expression of CCL2 and CXCL1 in astrocytes. The higher expression of CCL2 and CXCL1 in cultured astrocytes is in agreement with the observation that astrocytes are the main source of these chemokines [3]. We previously showed that intracerebroventricular administration of an ET_B_ agonist increased CCL2 and CXCL1 production in rat cerebral astrocytes [28]. In this study, treatment with ETs stimulated the production and release of CCL2 and CXCL1 in cultured astrocytes (Figures 1 and 4). The effect of ET receptor agonist and antagonists showed that the actions of ET-1 were mediated by ET_B_ receptors (Figures 2 and 3). From these findings, activation of astrocytic ET_B_ receptors is thought to stimulate CCL2 and CXCL1 production directly. Increased production of astrocytic CCL2 and CXCL1 was observed in nerve tissue damaged by brain ischemia and neurodegenerative diseases [4,6,36,37]. Brain ETs have been shown to be increased in several brain pathologies and regulate several pathophysiological responses of astrocytes, including the production of extracellular signaling molecules, through ET_B_ receptors [27]. Thus, the ET-induced chemokine production in cultured astrocytes suggests that ETs are one of the factors to stimulate CCL2 and CXCL1 production at the damaged nerve area.

### ETs decrease astrocytic CX3CL1 production

Differing from CCL2 and CXCL1, production of astrocytic CX3CL1 decreased following treatment with ETs (Figures 1 and 4), which was also mediated by ET_B_ receptors (Figures 2 and 3). CX3CL1 is relatively abundant in the brain, where sub-populations of neurons constitutively express the protein [1]. We found that cultured astrocytes had a comparably high level of CX3CL1 when compared to cerebral neurons (Table 1). As for the regulation of astrocytic CX3CL1, pro-inflammatory cytokines, such as tumor necrosis factor alpha (TNFα) and IFN-γ, stimulate its production in cultured astrocytes [38]. On the other hand, a negative-regulatory mechanism of constitutive CX3CL1 production was suggested by the finding that basal CX3CL1 production in human astrocytomas was reduced by tumor growth factor beta (TGFβ) [18]. The effects of ETs on CX3CL1 production indicate an involvement of ET_B_ receptors in the negative-regulation of astrocytic CX3CL1 production. Recently, Donnelly *et al*. [39] showed that expression of CX3CL1 decreased after spinal cord injury in mice, although its cellular sources were not identified. Thus, the negative regulation of astrocytic CX3CL1 by ETs may reflect the reduced CX3CL1 expression in damaged nerve tissues.

### Signal transduction mechanisms mediating the ET-induced chemokine production

Activation of astrocytic ET_B_ receptors stimulates several intracellular signal pathways, including PKC, intracellular Ca^2+^, and MAP kinases. The effects of ET-1 on astrocytic chemokine production were significant at 10 to 100 nM (Figure 1B), which concentrations of ET-1 activated signal mechanisms mediated by PKC, Ca^2+^ and MAP kinases [25,33-35]. The effects of signal transduction inhibitors (Table 2 and Figure 5) showed that different mechanisms mediate ET_B_ receptor signals to regulate astrocytic chemokine expression. In addition to the regulation of gene transcription, expression levels of CCL2, CXCL1 and CX3CL1 mRNA can be regulated by alteration of their stabilities [40-43]. The effect of ET-1 on CCL2 and CXCL1 mRNA expression was inhibited by actinomycin D (Figure 5A). Further examination showed that treatments with ET-1 did not affect the degradation rates of astrocytic CCL2 and CXCL1 mRNAs (Figure 5B). These results suggest that stimulation of transcription, rather than increases in mRNA stability, underlie ET-induced astrocytic CCL2 and CXCL1 expression. Both rat CCL2 and CXCL1 genes have recognition sequences for NFκB and SP1 on the 5′-promotor regions. Through these recognition sites, transcription of CCL2 and CXCL1 are cooperatively stimulated by NFκB and SP1 [44-47]. Agreeing with these findings, the inhibition of astrocytic CCL2 and CXCL1 expression by PDTC, SN50 and mithramycin suggests the involvement of both NFκB and SP1 in the effect of ET-1. MAP kinases, that is, ERK, JNK and p38, regulate the transcription activities of NFκB and SP1 in signal transduction pathways under several receptors. In astrocytes, activation of JNK and p38 was reported to stimulate NFκB [48,49]. We also found that ET-induced activation (phosphorylation) of SP1 was reduced by SP600125 in cultured astrocytes [see Additional file 2]. Thus, the inhibition of ET-induced CCL2 and CXCL1 expression by SP600125 and SB203580 may indicate that JNK and p38 mediate ET receptor signals to NFκB and SP1.

Differing from the effects on CCL2 and CXCL1 expression, the ET-induced decrease in CX3CL1 mRNA was inhibited by cycloheximide (Figure 5A). This result indicates a requirement of protein *de novo* synthesis for the effect of ETs on fractalkine expression. Moreover, Ca^2+^ chelation and PKC inhibition, but not MAP kinase inhibition, prevented the effects of ET-1. Rat CX3CL1 mRNA has AU-rich elements in the 3′ regions, where many regulatory proteins affecting mRNA stability bind [42]. As is reported in the regulatory mechanisms of some inflammatory factors [50], ET may stimulate the induction of regulatory proteins that destabilize CX3CL1 mRNA through Ca^2+^- and PKC-dependent signals.

### Pathophysiological significance of the ET-induced changes in astrocytic chemokine production

In nerve tissues damaged by brain insults and neurodegenerative diseases, astrocytes undergo a phenotypic change to reactive astrocytes and alter their ability to produce various chemokines [7]. By altering the production of astrocyte-derived chemokines, the pathophysiological response of the damaged brain is modulated. In brain pathologies, brain ETs increase in damaged tissues, which activate astrocytic ET_B_ receptors and induce reactive astrocytosis [25,26]. Accompanied with the conversion to reactive astrocytes, ETs modulate the production of various extracellular signaling molecules [27]. A major finding of the present study is that ETs had different actions on astrocytic chemokine production: ETs increased CCL2 and CXCL1, but decreased CX3CL1 production (Figures 1 and 4). The reciprocal regulation of astrocyte-derived chemokines would result in the possible modulation of chemokine-induced pathological brain responses by ETs. In the brain, receptors for CCL2, CXCL1 and CX3CL1 are expressed in vascular endothelial cells, neurons and microglia [1]. CCL2, CXCL1 and CX3CL1 all stimulate the proliferation and migration of vascular endothelial cells [51-53], which indicates that these chemokines have similar actions on neovascularization after brain injuries. The function of these chemokines on neuronal cells is controversial. While CX3CL1 showed a neuroprotective effect [9,11], CCL2 and CXCL1 were reported to be detrimental [8] or protective [10,12] on neuronal cells. Thus, the possible significance of ET-induced astrocytic chemokine production would be difficult to discuss in view of the function of neurons and vascular endothelial cells.

On the other hand, the action of CX3CL1 opposes that of CCL2 and CXCL1 in the regulation of microglial function. CCL2 and CXCL1 caused the activation of cultured microglia and stimulated the production of proinflammatory molecules [19,54]. Inhibition of CCL2 signals attenuated microglial activation and pro-inflammatory cytokine production in animal models of brain injury [19,55]. Pro-inflammatory cytokine production and migration in cultured microglia were stimulated by CXCL8/IL-8, a human homologue of rat CXCL1 [14,15]. In contrast, CX3CL1 attenuated microglial activation and proinflammatory cytokine production *in vitro* and *in vivo*[16,17]. Mice lacking CX3CL1 receptors showed enhanced activation of microglia in response to lipopolysaccharide [13], indicating a repressive role of CX3CL1 in microglial function. Considering the different actions among CCL2, CXCL1 and CX3CL1 on microglia, the reciprocal regulation of astrocytic chemokine production by ETs may have a pathophysiological significance in the induction of activated microglia. Induction of activated microglia promotes the neuroinflammatory response and results in the aggravation of neuronal degradation. Thus, the increase in ETs after brain insults and neurodegenerative diseases may show a detrimental action on the damaged brain through microglial activation induced by altered astrocytic chemokine production.

## Conclusions

In this study, activation of ET_B_ receptors altered the production of CCL2, CXCL1 and CX3CL1 in cultured astrocytes. Because astrocytes are a main source of brain chemokines in neurological disorders, alterations of astrocytic chemokine production affect several responses of the damaged brain. Thus, ET-induced alterations of astrocytic chemokine production indicate a pathophysiological significance of astrocytic ET_B_ receptors.

## Abbreviations

ANOVA: analysis of variance; BAPTA/AM: 1,2-bis(2-aminophenoxy)ethane N,N,N',N'-tetraacetic acid acetoxymethyl ester; CINC-1: cytokine-induced neutrophil chemoattractant-1; DMSO: dimethyl sulfoxide; EGTA: ethylene glycol tetraacetic acid; ELISA: enzyme-linked immunosorbent assay; ET: endothelin; G3PDH: glyceraldehyde-3-phosphate dehydrogenase; GFAP: glial fibrillary acidic protein; IFN: interferon; IL: interleukin; MAP: mitogen-activated protein; MCP-1: monocyte chemoattractant protein-1; MEM: minimal essential medium; NFκB: nuclear factor-KappaB; PDTC: pyrrolidine dithiocarbamate; PKC: protein kinase C; PLSD: protected least significant difference; PCR: polymerase chain reaction; RT: reverse transcriptase; SDF-1: stromal cell-derived factor-1; SEM: standard error of the mean; TGF: tumor growth factor; TNF: tumor necrosis factor.

## Competing interests

The authors declare they have no competing interests.

## Authors’ contributions

YK and MS participated in the design of this study and the preparation of the manuscript. YK, MK, TS, MK and RK preformed the research work. All authors read and approved the final manuscript.

## Supplementary Material

Additional file 1 Effects of signal transduction inhibitors on CCL2, CXCL1 and CX3CL1 mRNA levels in cultured astrocytes. Click here for file

Additional file 2 Effects of MAPK inhibitors on the ET-induced phosphorylation of SP1. Click here for file
