# Supplementary material for: Different actions of endothelin-1 on chemokine production in rat cultured astrocytes: reduction of CX3CL1/fractalkine and an increase in CCL2/MCP-1 and CXCL1/CINC-1
Source: J Neuroinflammation. 2013 Apr 30;10:51. doi: 10.1186/1742-2094-10-51 (PMC3675376; doi:10.1186/1742-2094-10-51)
Supplement: Additional file 1 — Effects of signal transduction inhibitors on CCL2, CXCL1 and CX3CL1 mRNA levels in cultured astrocytes. [file 1742-2094-10-51-S1.docx]

Additional file 1.

Effects of signal transduction inhibitors on CCL2, CXCL1 and CX3CL1 mRNA levels in cultured astrocytes

Ratio of chemokine/G3PDH mRNA copy numbers

(% of none)

CCL2/MCP-1 CXCL1/CINC-1 CX3CL1/fractalkine

none 100.0 ± 38.8 (6) 100.0 ± 7.7 (6) 100.0 ± 30.8 (8)

30 μM BAPTA/ 0.5 mM EGTA 124.6 ± 48.5 (7) 85.2 ± 31.9 (7) 104.1 ± .38.8 (5)

10 nM Staurosporine 83.9 ± 23.7 (7) 89.3 ± 8.3 (7) 103.3 ± 32.0 (6)

100 μM PDTC 113.7 ± 27.3 (7) 91.4 ± 39.0 (7) 102.7 ± 53.8 (6)
10 μM SN50 111.8 ± 31.2 (6) 84.8 ± 9.7 (6) 95.3 ± 24.0 (5)

500 nM Mithramycin 77.9 ± 24.8 (8) 130.2 ± 5.9 (8) 75.6 ± 24.0 (5)

50μM PD98059 84.0 ± 23.0 (8) 117.7 ± 46.1 (8) 122.9 ± 9.6 (6)

20μM SB203580 118.9 ± 31.2 (6) 131.2 ± 11.3 (6) 105.3 ± 45.1 (6)

1 μM SP600125 87.9 ± 23.6 (8) 95.3 ± 39.9 (8) 122.1 ± 53.8 (6)

Cultured astrocytes were treated with the signal transduction inhibitors at the concentrations indicated. For determination of mRMA levels of CCL2, CXCL1 and CX3CL1, total RNA was extracted after 1.5 and 6.5 hours, respectively. The copy numbers of CCL2, CXCL1 and CX3CL1 mRNA was normalized to G3PDH. Results are means ± SEM and the numbers of experiments are in parentheses.
