# Supplementary material for: Different actions of endothelin-1 on chemokine production in rat cultured astrocytes: reduction of CX3CL1/fractalkine and an increase in CCL2/MCP-1 and CXCL1/CINC-1
Source: J Neuroinflammation. 2013 Apr 30;10:51. doi: 10.1186/1742-2094-10-51 (PMC3675376; doi:10.1186/1742-2094-10-51)
Supplement: Additional file 2 — Effects of MAPK inhibitors on the ET-induced phosphorylation of SP1. [file 1742-2094-10-51-S2.pdf]

Additional file 2.

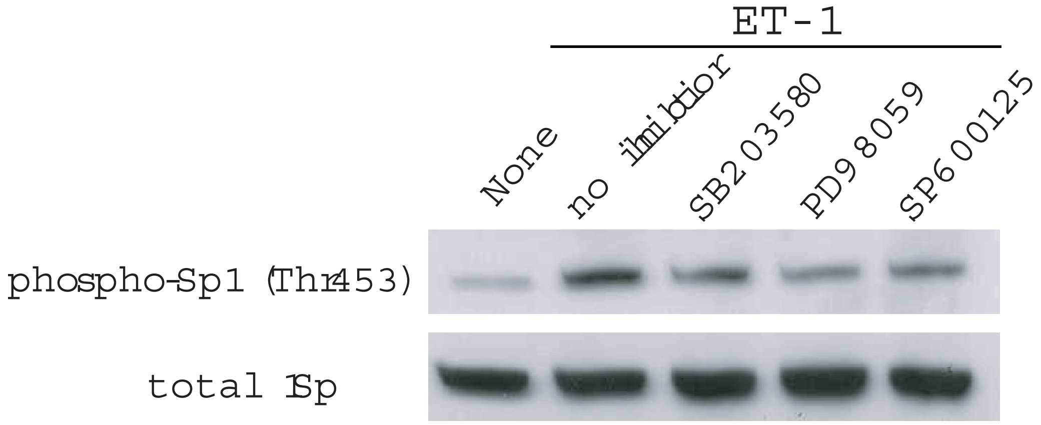

Effects of MAPK inhibitors on the ET-induced phosphorylation of Sp1. After cultured astrocytes were treated with MAPK inhibitors for 30 min, cells were further incubated with 100 nM ET-1 for 30 min. Preparation of cell lysate and SDS-PAGE were performed as described before (Koyama et al., J Neurochem. 90:904-912. (2004)). Blotted membranes were probed with rabbit antibodies against phospho-T453-Sp1 (Abcam, Tokyo, Japan) and Sp1 (Abcam), and then, incubated with a peroxidase-conjugated secondary antibody.

Concentrations of MAPK inhibitors are as follows: PD98059; 50 μM, SB203580; 20μM, SP600125; 1 μM.
